# Oesophagoscopy

**Published:** 1907-01-05

**Authors:** W. Stuart Low

**Affiliations:** Lecturer and Joint Teacher of Practical Otology, Polyclinic College


### HOSPITAL CLINICS.

### ŒSOPHAGOSCOPY.

### Demonstration to Practitioners at the Central London Throat, Nose, and Ear Hospital.

By W. STUART LOW, F.R.C.S.(Eng.), Lecturer and Joint Teacher of Practical Otology, Polyclinic College.

MR. STUART LOW said that the direct examination of the œsophagus by means of straight metal tubes introduced from above and pushed along to various depths had not been systematically studied nor practised in this country. On the Continent, and more particularly in Germany, however, this matter has been gone into with the usual Teutonic energy and thoroughness, with the result that the Germans are *facile princeps* in this interesting, instructive, and invaluable field of clinical investigation. Why we should lag behind was rather difficult to explain. It might be, but if so it was quite incorrect, that œsophagoscopy was considered by British medical authorities as of little use in its practical application. Just as in the case of submarines, airships, and balloons, however, a powerful advocacy and insistence on their great practical utility in the lay press had had the result of thoroughly awakening us to the urgent necessity of adopting them, so it has become imperative that our more energetic and untrammelled medical press should perform the same freelance-like work of forcing an adequate recognition of the great importance of œsophagoscopy as an aid in diagnosis and treatment.

Mr. Stuart Low remarked that he would only say a word here on the perfect safety of direct inspection of the gullet by means of the long single tube in well-trained hands. He mentioned this particularly because there was an idea abroad among students and medical men that the method was dangerous, and he had often been questioned by his pupils as to the great risks run of perforation of the œsophagus.

Before the introduction of the single long metal inspecting tube of Mikulicz, when jointed tubes, that could be subsequently straightened, as used originally by Kelling and Stork were used, there was considerably more danger, but now the likelihood of any serious complication was reduced to a minimum in skilled and practised hands.

Killian, like his predecessor in the chair of Laryngology in the University of Freiberg, has done much to develop and popularise this method of œsophageal examination.

The best instruments at present in use are those improved by Killian—namely, the long metal tubes and special lamp fixed at the proximal end for direct illumination when the Kerstein lamp, worn on the forehead, was not being used. For class demonstration purposes direct illumination was the best, as all the members of the class could easily file past and successively look down the rigid metal tube and thus inspect the œsophageal interior. For illumination either a storage battery or the street or house current could be employed. The metal tubes vary in length from 19 to 50 cm., and in diameter from 1 to 1.5 cm. The parts of the œsophagus most commonly diseased are the beginning, the region of the tracheal bifurcation situated 26 cm. from the incisor teeth and the termination.

#### THE METHOD.

One of the most important and essential practical points in the successful carrying out of œsophagoscopy consists in the preparation of the patient. To suddenly commence the direct inspection of the œsophagus without first properly preparing the patient is only to court failure.

The clothes about the neck and chest should be freely loosened so that the neck can be straightened and the head thrown back without the patient feeling any constriction, and so that the chest can be expanded and respiration carried on quite freely.

The stomach should be empty so that the patient should not have partaken of food nor drink for some time previously to the examination. Vomiting sometimes arises, particularly when the tube comes into contact with the cardiac aperture and the stomach contents flow into the tube. These contents should be mopped out with cotton wool or sucked up by a pump. If the patient is in the horizontal position the vomited substance will be got rid of by lowering the patient's head or raising the foot of the operating table.

It may sometimes be advantageous to perform œsophagoscopy in the horizontal position on the operating table and under complete anæsthesia, but this is quite exceptional. The usual position for inspection is the sitting position, with the head efficiently supported and thrown well back, so as to straighten the passages as much as possible. Killian is very careful to cocainise the pharynx and upper part of the œsophagus thoroughly. He does this by means of a long malleable curved wool carrier and with the wool well saturated with a 10-per-cent. solution of cocaine these mucous surfaces are again and again mopped over at intervals of ten minutes. In this way complete local anæsthesia is established, and the subsequent procedure very greatly facilitated.

When studying under Killian some months ago at Freiberg the assiduity with which he applied the cocaine previous to passing the instrument was much impressed upon me as an essential feature of his method and success, and in his clinic cocainisation is carried out with the aid of the laryngeal mirror.

If the patient wears artificial teeth they ought to be removed, and it was pointed out at this demonstration that people with edentulous jaws were the best to commence practising upon, as the straight rigid tube can be more easily passed in the absence of the upper incisor teeth.

It is very important that œsophagoscopy should always be preceded by a very careful examination of the œsophagus with graduated bougies in order to discover the seat of the disease and to be enabled to choose the right length of tube. Should this proceeding be negative, then the longest tube must be employed, so as to reach the cardia, and by gradually withdrawing the tube every portion of the gullet can be successively illuminated.

The metal tube having been warmed and lubricated has first passed into it a hard rubber sound obliquely rounded at the tip, and this occludes its cavity and prevents it becoming filled with saliva and mucus. The base of the tongue is gently drawn forward and downwards by means of the left hand, and with the right hand the instrument is made to glide over the tongue and epiglottis and on to the posterior wall of the pharynx. This is accomplished rather rapidly, and somewhat forcibly, and in this way the tube overcomes the normal resistance caused by the larynx pressing against the vertebral column and the contraction of the inferior constrictor of the pharynx, and usually glides on unhindered into the œsophagus. If the instrument is now directed properly, it is exceptional to encounter any marked muscular resistance. Should it become caught, however, or refuse to advance far with slight force, then the central hard rubber sound should be at once withdrawn and Casper's or Killian's lamp pushed into position and fixed over the orifice of the metal tube, and by this good illumination the tube can be kept in the centre of the lumen of the œsophagus and pushed towards the cardia with facility, should no abnormal obstruction exist. This precaution is particularly necessary should advanced carcinoma, ulceration processes, or any internal constriction or irregularity be present, to avoid perforation of the œsophageal wall. Having reached the cardiac end, the tube is then slowly and carefully withdrawn, and the receding walls systematically and successively inspected for the detection of irregularities, ulcerations, or congested spots or other departures from the normal. Should saliva and mucus occlude the view it should be mopped up by means of cotton wool or small pieces of sponge fixed on proper carriers.

In particularly difficult cases the tube is best introduced by being passed over an English woven œsophageal bougie, which is first inserted and which exactly fits the metal tube and acts as an efficient guide—this being subsequently withdrawn when the metal tube is in position.

When sufficient manipulative skill has been acquired, the patent long metal tube can be introduced right down to the cardia without the aid of either the bougie or the hard rubber sound.

The anatomical course of the œsophagus serves to explain some of the hindrances and difficulties of introduction. It is to be particularly noted that in the lower portion of the thoracic œsophagus it is situated towards the right and posteriorly, and that it is only on reaching the diaphragmatic orifice that it turns forwards and to the left.

Mr. Stuart Low demonstrated the passage of the long metal tube to the students in the case of a female patient, 65 years of age, who first came to the clinic a fortnight ago. The woman applied for relief because of difficulty in and coughing on swallowing liquids, and more particularly warm liquids. Examination of the larynx revealed complete fixation of the right vocal cord in the cadaveric position. The voice was not at all affected, although easily tired after speaking for a short time. The absence of hoarseness was explained by the fact that the unaffected vocal cord readily passed across and approximated well to the fixed cord on vocalisation. Some hard glands could be made out on the right side in the superior deep cervical lymphatic group. The diagnosis, as revealed by the œsophagoscope, was a unilateral spot of malignant disease on the right side of the œsophagus implicating the recurrent laryngeal, and thus causing paralysis of the muscles moving the vocal cord and impairment of the sphincters of the larynx. This would also explain the greater difficulty and embarrassment in the swallowing of warm as compared with cold liquids, since the cold fluids would tend to stimulate the paretic sphincters, while the warm would not.

Another case demonstrated was also that of a female patient, aged 56, who came to the clinic complaining of loss of power to swallow solids and almost complete obstruction for liquids. Laryngological inspection showed the left vocal cord very impaired in its movements, but no other physical sign. On attempting to pass a large-sized œsophageal bougie, however, it was quite impossible to get beyond the region of the cricoid cartilage, six inches from the teeth, and the end of the bougie was found coated with blood. Inspection by means of the tube showed a ring carcinoma at the upper part of the œsophagus, and when withdrawn this could not only be plainly seen, but its surface and consistency could be made out by means of a probe passed down through the tube, and a fine india-rubber tube could be guided over the growth, and in this manner feeding carried out successfully.

Mr. Stuart Low told the class of a very important and interesting case that he had seen in Professor Killian's clinic. A woman had suffered so much pain on taking food that for some months she had been unable to eat, and in spite of much medical treatment had become very thin and lost much in weight. Inspection of the gullet by the long tube showed a very red ring of superficial ulceration near the cardia; this was mopped over every second day through the tube with a solution of silver nitrate, with the result that in a few weeks the patient was completely restored to her usual health.

*Foreign bodies.*—The œsophagus extends from the cricoid cartilage to the entrance into the stomach opposite the tenth dorsal vertebra. It is from nine to ten inches in length, and a little over half an inch in diameter.

There are three narrow parts in its course, one at its commencement, one $2\frac{3}{4}$ inches below that point, and a third where it passes through the diaphragm. Foreign bodies, when swallowed, are most likely to lodge at one of these natural constrictions. It is most important to remove anything lodging in this passage as soon as possible, since, if allowed to remain, a foreign body is apt to lead to ulceration that may open into adjacent parts. In this way a coin lodged in the œsophagus has caused death by ulcerating into the aorta. Removal can be best accomplished by special forceps introduced through the œsophageal tube. Mr. Stuart Low showed these forceps and demonstrated the method of using them.

